# Can surfactants affect mortality and morbidity in term infants with respiratory failure?

**DOI:** 10.55730/1300-0144.5523

**Published:** 2022-10-22

**Authors:** Senem ALKAN ÖZDEMİR, Özlem YILMAN, Şebnem ÇALKAVUR, Tülin GÖKMEN YILDIRIM

**Affiliations:** 1 Division of Neonatology, Dr. Behçet Uz Child Disease and Pediatric Surgery Training and Research Hospital, University of Health Sciences Turkey, İzmir, Turkey; 2 Department of Pediatrics, Division of Neonatology, İzmir Faculty of Medicine, University of Health Sciences Turkey, İzmir, Turkey; 3 Department of Pediatrics, Dr. Behçet Uz Child Disease and Pediatric Surgery Training and Research Hospital, University of Health Sciences Turkey, İzmir, Turkey

**Keywords:** Morbidity, mortality, respiratory failure, surfactant, term infant

## Abstract

**Background/aim:**

We aimed to discuss term infants who are given surfactant due to respiratory disorder according to the underlying etiology, the dose of surfactant administration, and the need for repeated surfactant administration.

**Materials and methods:**

In this retrospective study infants hospitalized in the 4th level neonatal intensive care unit during January 2019 and December 2021 and administered surfactant due to respiratory distress were included. Term infants given surfactant due to respiratory failure were included in the study through the data recording system. The number of surfactant doses, indications for administration, mortality, duration of hospitalization, intubation time, and inotrope use were recorded in the infants included in the study.

**Results:**

During the two-year period, 1250 infants were hospitalized in our neonatal intensive care unit. Of those, 56 infants received surfactant replacement therapy for severe respiratory failure. There were 30 infants with pneumonia, 4 infants with meconium aspiration syndrome (MAS), and 22 infants with transient tachypnea of the newborn (TTN). It was seen that single-dose administration was higher in patients with TTN (p = 0.01), while multiple-dose surfactant administration was more common in patients with MAS, resulting in a statistical difference (p = 0.02). Mortality was lower, especially in cases given early surfactant administration and this situation was statistically significant (p < 0.001). Duration of intubation was 5.05 ± 4.7 days in early surfactant administration group and 8.0 ± 6.1 days in late surfactant administration group. This difference was statistically significant (p = 0.04). While early surfactant application was statistically higher in the TTN group (p = 0.007), late surfactant application was statistically higher in the pneumonia group (p = 0.001).

**Conclusion:**

Despite the difference on administration time and repeat dose interval due to etiology, surfactant treatment is improving the respiratory distress of term infants.

## 1. Introduction

Respiratory distress syndrome (RDS) is the most common respiratory failure condition in preterm infants. This situation increases as the gestational week of the infant decreases. However, respiratory distress is a very important problem not only for preterm infants but also for term infants [1]. Although there are many risk factors for the etiology of respiratory distress in newborn infants, cesarean delivery, which has increased in recent years, poses a very important risk [2]. There is an inversely proportional relationship between respiratory distress and gestational age in term newborn infants and the incidence of respiratory distress decreases as the gestational week progresses [2]. There are many reasons that cause respiratory distress in the neonatal period, the most common reason of respiratory distress is due to pulmonary causes. When the pulmonary causes of respiratory distress in term infants are examined, causes such as transient tachypnea of the newborn (TTN), neonatal pneumonia, pneumothorax, and pulmonary hypertension are the leading causes [3]. In these cases, the surfactant may be needed, similar to respiratory distress syndrome in preterm infants. In this study, we aimed to discuss term infants who are given surfactant due to respiratory disorder according to the underlying etiology, the dose of surfactant administration and the need for repeated surfactant administration.

## 2. Materials and methods

This is a retrospective and descriptive study, and the study was conducted by Health Sciences University İzmir Dr. Behçet Uz Pediatrics and Surgery Training and Research Hospital. Infants hospitalized in the 4th level neonatal intensive care unit and administered surfactant due to respiratory distress were included. Our unit is the largest neonatal intensive care unit in the Aegean region and includes 58 beds. It serves around 1500 newborn infants annually.

Term infants hospitalized due to respiratory distress for a period of two years (2019 January to 2021 December) were included in the study. In newborns, tachypnea (respiratory rate > 60/min) and tachycardia (peak heart rate > 160/min), cyanosis, nasal flaring, apnea/dyspnea, groaning, and chest wall retractions are defined as respiratory failure [4]. In accordance with this definition, term infants given surfactant due to respiratory failure were included in the study through the data recording system. The number of surfactant doses, indications for administration, mortality, duration of hospitalization, intubation time, and inotrope use were recorded in the infants included in the study. Cases with major structural congenital anomaly, cardiac problem, diagnosed with metabolic disease in the follow-up and genetic malformation were not included.

Diagnostic criteria for the lung diseases were as follows: [1] Transient tachypnea of the newborn: Transient tachypnea of the newborn is a diagnosis based on clinical and radiological findings. Often, the diagnosis of TTN is made by excluding other diagnoses such as RDS, pneumonia, and pneumothorax [5]. If the signs of respiratory distress increase, the fractionated inhaled 0_2_ (FiO_2_) requirement exceeds 40%, there are abnormal chest X-ray findings, or if the baby does not improve even after two h, infant should be hospitalized in the neonatal intensive care unit [2,6]. Meconium aspiration syndrome (MAS): It is a picture that progresses with varying degrees of respiratory distress and hypoxic respiratory failure due to inhalation of meconium and amniotic fluid during fetal life or birth. Meconium aspiration syndrome is defined as the presence of respiratory distress in the first two h of life, the need for oxygen support, and the absence of any other explainable airway, lung, or congenital heart malformation [7]. Respiratory distress in an infant born through meconium-stained amniotic fluid whose respiratory and radiological signs cannot be otherwise explained [2]. Pneumonia: Pneumonia is the inflammatory process of the lung. It is diagnosed with clinical, radiological and microbiological findings. Because pneumonia findings are nonspecific, patients with sudden onset respiratory distress should be evaluated for pneumonia with complete blood count, acute phase reactants, culture and chest X-ray, including complete sepsis evaluation [8].

Pulmonary hypertension was defined as elevated right ventricular and pulmonary artery pressure leading to right to-left ductal or foramen shunt detected by echocardiography, a routine procedure in our NICU for the patients suffering from severe respiratory failure. Surfactant was prepared according to the methods described in a published trial and administrated at a dose of 100 mg/kg [9]. Poractant alfa (Chiesi, Italy) was used for all patients. Surfactants were administered if the infants had fractionated inhaled oxygen levels higher than 60% or persistent low saturation levels (<85%). Surfactant administration in the first 4 h was accepted as early, surfactant application after 24 h was considered as late surfactant treatment. Two or more surfactant administrations were considered as multiple dose surfactant application. The primary outcome was mortality and secondary outcome was duration of the oxygen support according to early surfactant administration. The research protocol was fully approved by local ethics committee.

Statistical data were analyzed by using the Statistical package for the Social Sciences (SPSS) 15.0 software (SPSS Inc., Chicago, IL). Continuous variables were compared by using Mann-Whitney U-test. Categorical variables were analyzed by chi-square test or Fisher’s exact test. A p-value of <0.05 was accepted as statistically significant.

## 3. Results

During the two-year period, 1250 infants were hospitalized in our neonatal intensive care unit (NICU). Of those, 56 term infants received surfactant replacement therapy for severe respiratory failure. Demographic characteristics of the patients are given in Table 1. There were 30 infants with pneumonia, 4 infants with MAS and 22 infants with TTN. Pulmonary air leak syndrome was not seen whereas pulmonary hemorrhage occurred in one infant with MAS. Two infants with TTN died and 6 infants died due to pneumonia. One infant with MAS died. We did not administer surfactant to any patients due to ARDS, pulmonary hemorrhage. Infants with congenital diaphragmatic hernia and major congenital anomalies were excluded from the trial. Pulmonary hypertension was developed in 10 infants but they did not require inhale nitric oxid treatment. Nine infants died with a mortality rate of 16%. Mortality rate was higher in patients with MAS.

A single dose of surfactant was administered to 73.2% of the infants included in the study. When infants were evaluated according to single and multiple dose surfactant administration, it was seen that single dose administration was higher in infants with TTN (p = 0.01), while multiple-dose surfactant administration was more common in infants with MAS, resulting in a statistical difference (p = 0.02). The rate of inotropic agent use was 66.6% in the group given multiple doses and it was statistically significant (p = 0.006). The duration of mechanical ventilation was found to be longer in infants given multiple doses of surfactant (p = 0.001). It is shown in detail in Table 2.

In the evaluation of mortality and surviving infants, mortality was higher in infants using inotropes (p = 0.04). It was determined that two infants died due to TTN and these two infants received late surfactant treatment. It was observed that 15 of the infants diagnosed with pneumonia received late surfactant treatment, and all of the infants hospitalized due to MAS were treated with early surfactant.

When the surfactant was evaluated according to the time of administration, it was seen that mortality was lower especially in cases given early and this situation was statistically significant (p < 0.001). Similarly, the duration of stay in the mechanical ventilator was found to be shorter in infants treated with early surfactant (p = 0.04). It is shown in detail in Table 3.

## 4. Discussion

In this trial we aimed to investigate the surfactant usage in term respiratory disorders. We found that surfactant treatment is an important point for the term respiratory failure. Fifty-six infants received surfactant replacement therapy for severe respiratory failure and pneumonia is the most common surfactant treatment indication at the NICU admission. We also found that single dose surfactant administration was higher in TTN, and mortality was lower especially in cases given early surfactant application.

Respiratory distress syndrome is a major cause of morbidity and mortality in preterm neonates [1,10]. Pulmonary surfactant deficiency is the primary cause of respiratory distress syndrome. It affects 40%–50% of the neonates born before 32 weeks of gestation and results in high morbidity and mortality [11]. Surfactant-replacement therapy is a life-saving treatment for preterm infants with the introduction of surfactant treatment, especially premature mortality has decreased significantly [12].

Transient tachypnea of newborn is one of the most common causes of neonatal respiratory distress. Increased fluid volume in the airways, uterine contractions, pulmonary immaturity, and genetic predisposition are involved in the pathophysiology of TTN. Vaginal or cesarean delivery without medical indication should be avoided before the 39th gestational week [13]. Although the gestational age of all infants included in our study is 38 weeks and above, the high cesarean section ratio creates a very important risk for TTN. Another important point is that the patients we followed up for TTN needed a single dose of surfactant treatment. Only 2 term infants with TTN needed multiple dose of surfactant treatment. This led to a statistical difference. It has been suggested that there may be problems related to the production and function of surfactant, especially in the first hours of life. In a study conducted with this hypothesis, 42 infants with TTN were evaluated and they found that low lamellar body counts are associated with decreased surfactant function, suggesting that prolonged disease is associated with surfactant abnormalities [14]. A similar study was done by Fiori et al. [15] and deficiency or dysfunction of the surfactant system is involved in the majority of cases of respiratory distress in near term and possibly term infants. In another study which 544 infants were born to via elective cesarean delivery examined and oral fluid samples were obtained in the delivery room immediately after birth, and gastric fluid was collected within the first hour of life [16]. They suggest that TTN was associated with surfactant dysfunction. In parallel with these studies, we found the frequency of surfactant application was high in TTN and compared whether early/late single/multiple dose application had an effect, unlike other studies. In line with the data of our study, early administration of surfactant may be considered in cases of increased respiratory distress in patients hospitalized in the NICU due to TTN during follow-up.

Meconium aspiration syndrome is caused by inflammation in the airways and can cause severe obstructive pulmonary disease with significant increase in airway resistance, airway obstruction leading to emphysema, and/or atelectasis (if the involved airway is completely obstructed) [17]. The disease is usually pulmonary involvement due to widespread atelectasis and over-ventilated areas, indicating a high risk of air leaks [17]. Also, meconium inactivates surfactant and thus can cause an acute respiratory distress syndrome such as restrictive lung disease, which can be managed at higher rates or with high frequency ventilation and/or surfactant replacement therapy [18]. Typically, meconium aspiration often results in increased lung resistance. The combination of impaired airway mechanics combined with surfactant dysfunction creates a nonhomogeneous lung disease, complicating the management of severe MAS [19]. In the Cochrane meta-analysis, it was stated that the administration of surfactant in infants with MAS had no effect on mortality, but the studies were not homogeneous [20]. It was stated that the application of surfactant in these infants reduced the severity of respiratory disease and reduced the course to extracorporeal membrane oxygenation [20]. Although surfactant dysfunction is often present at the start of invasive respiratory support, conventional mechanical ventilation may further compromise its function. Therefore, it is vital to apply it in the early stages of the disease. In our study, we found that multiple-dose surfactant applications were high in infants with MAS. Although the number of cases is small, multiple-dose surfactant treatment may be necessary in MAS, and this is often life-saving.

Pneumonia is the inflammatory process of the lung. It has an important place in neonatal infections and is an important cause of morbidity and mortality in developing countries. In pneumonia, there is usually increased permeability of both the endothelial and epithelial barriers, increasing the influx of plasma proteins into the alveolar space [21]. At the same time, pulmonary surfactant is an important part of the host defense against respiratory infections. Substances secreted directly by bacteria impair surfactant synthesis directly and indirectly through pulmonary type 2 epithelial cells. The interaction of bacteria or endotoxin with the secreted surfactant causes changes in the physical (i.e. density and surface tension) properties of the surfactant. Changes in surfactant have a detrimental effect on lung function, characterized by significant decreases in total lung capacity, static compliance, diffusion capacity and arterial partial oxygen pressure, and a significant increase in mean pulmonary artery pressure. In addition, reduced surfactant concentrations or an altered surfactant composition can cause anatomical changes commonly seen in pneumonia, such as pulmonary edema, hemorrhage, and atelectasis [22].

A 2012 Cochrane meta-analysis found no evidence from randomized controlled trials to support or disprove the efficacy of surfactant in late preterm and term infants with proven or suspected bacterial pneumonia, but it has been clearly demonstrated that surfactant is affected in the case of pneumonia [23]. In our study, the need for surfactant was mostly required in infants hospitalized for pneumonia. Both single and multiple doses of surfactant were needed in these infants. However, surfactant use is higher in the late period because these infants usually have late-onset pneumonia. There are small studies showing that surfactant treatment may be needed and even multiple dose applications may reduce mortality in patients who are hospitalized in the NICU with a pneumonia clinic and whose general condition deteriorates [24,25]. Therefore, we think that the administration of surfactant in newborns with pneumonia is very important, especially in cases with a poor clinical course. In this study, surfactant administration time was mostly after 24 h in infants who were administered surfactant for pneumonia, which we attribute to the rapid deterioration of these patients. Close follow-up of infants who are at risk for pneumonia is very essential for term infants with a respiratory disorder.

The small number of patients in our study is the most important limitation. In studies involving large patient populations, we can get more ideas about surfactant administration, time of administration and dose frequency, especially in term infants presenting with respiratory distress.

In conclusion, this study is the first to investigate whether there is a difference in the etiology, dose frequency, and time of surfactant administration in term infants hospitalized in the NICU due to respiratory distress and treated with surfactant.

## Figures and Tables

**Table 1:** Demographic characteristics of the infants included in the trial.

Gestational week (wk)*	38.0 ± 1.14
Birth weight (g)*	2966 ± 500
Male sex (%)	30 (53.5)
Cesarean section (%)	48 (85.7)
Primiparous pregnant women (%)	10 (17.8)
Single dose surfactant (%)	41 (73.2)
Multiple doses surfactant (%)	15 (26.7)
Mortality (%)	9 (16)
Early surfactant administration (%)	39 (69.6)
Number of surfactant doses *	1.4 ± 0.75
Postnatal age of surfactant treatment (hour)*	10 ± 12

* As presented mean ± SD.

**Table 2:** Evaluation of infants according to the number of surfactant doses.

	Single dose (n = 41)	Multiple doses (n = 15)	p
Gestational week (wk)*	37.9 ± 1.0	38.2 ± 1.3	0.29
Birth weight (g)*	2928 ± 491	3070.6 ± 528	0.35
Male sex (%)	22 (53)	8 (53)	1.00
Cesarean section frequency (%)	36 (87)	12 (80)	0.66
TTN (%)	20 (48)	2 (13)	**0.01**
Pneumonia (%)	20 (48)	10 (66)	0.23
MAS (%)	1 (2)	3 (20)	**0.02**
Use of inotropes (%)	11 (26.8)	10 (66.6)	**0.006**
Mortality (%)	5 (12)	4 (26.6)	0.22
Duration of hospitalization (day)*	19.6 ± 13.9	23.8 ± 15.8	0.28
Duration of intubation (day)*	5.17 ± 5.5	8.0 ± 3.9	**0.001**

* As presented mean ± SD.

TTN: Transient tachypnea of the newborn

**Table 3:** Evaluation of surfactant by time of administration.

	Early administration (n = 39)	Late administration (n = 17)	p
Mortality (%)	0 (0)	4 (23.5)	**<0.001**
Duration of hospitalization (day)*	19.0 ± 12.6	24.7 ± 17.7	0.30
Duration of intubation (day)*	5.05 ± 4.7	8.0 ±6.1	**0.04**
Use of inotropes (%)	15 (38)	6 (35)	1.0
MAS (%)	4 (10)	0 (0)	0.17
Pneumonia (%)	15 (38)	15 (88)	**0.001**
TTN (%)	20 (51)	2 (11)	**0.007**

* As presented mean ± SD.
